# Effectiveness of Low Glycemic Index Diet Consultations Through a Diet Glycemic Assessment App Tool on Maternal and Neonatal Insulin Resistance: A Randomized Controlled Trial

**DOI:** 10.2196/12081

**Published:** 2019-04-18

**Authors:** Yi Zhang, Liping Wang, Wenhong Yang, Dayan Niu, Chunying Li, Liling Wang, Ping Gu, Yingqian Xia, Ying Shen, Juhua Yan, Qian Zhao, Kai Mu, Weili Yan

**Affiliations:** 1 Children's Hospital of Fudan University Shanghai China; 2 International Peace Maternity & Child Health Hospital of China Welfare Institute Shanghai China; 3 Kunshan Maternity and Child Care Center Kunshan China

**Keywords:** glycemic index, overweight, pregnancy, insulin resistance, randomized controlled trial

## Abstract

**Background:**

Low glycemic index (LGI) diet has shown to be effective in reducing maternal and neonatal complications in high-risk pregnancies.

**Objective:**

This trial aimed to examine the effectiveness of individualized LGI diet consultations based on the accurate diet glycemic load (GL) assessment tool on maternal and neonatal insulin resistance levels and diet behavior changes in overweight and obese pregnant women.

**Methods:**

Overweight and obese pregnant women were recruited before 16 weeks of gestation and randomized to the LGI diet arm or the control arm. All participants received standard dietary education according to the Chinese Dietary Guide for Pregnant Women. In the intervention arm, additional individualized dietary GL assessments were performed using an app and instructions of lowering diet glycemic index (GI) to achieve LGI diet were provided by a clinical dietitian at early, middle, and late gestation. Primary outcomes were serum insulin at late gestation, incidence of gestational diabetes mellitus (GDM) for mothers, and cord blood C-peptide level of neonates.

**Results:**

In total, 400 subjects were randomized and received different interventions. There were no significant differences in maternal serum insulin levels (13.2 [9.3−13.2] uU/mL vs 12.4 [10.5−12.4] uU/mL), incidence of GDM (45 [22.5%] vs 43 [21.5%]), or cord blood C-peptide levels (mean 0.9ng/mL [SD 0.7] vs mean 0.8ng/mL [SD 0.6]) in the intervention group compared with the controls. The diet GI at late gestation was similar (mean 63.2 [SD 10.4] vs mean 64.3 [SD 10.4]), whereas greater diet fiber intake was observed in the intervention group (mean 11.6 grams [SD 8.0] vs mean 9.0 grams [SD 5.6]; *P*=.006). Adherence measurements did not significantly differ between 2 groups.

**Conclusions:**

Individualized LGI diet consultations for overweight and obese pregnant women failed to make a significant difference in maternal or neonatal insulin resistance compared with the standard gestational diet consultation.

**Trial Registration:**

ClinicalTrials.gov NCT01628835; http://clinicaltrials.gov/ct2/show/NCT01628835 (Archived by WebCite at http://www.webcitation.org/77LHgWP0k)

## Introduction

### Background

The prevalence of overweight and obesity is increasing globally [1,2]. Data from China Chronic Disease and Risk Factor Surveillance survey indicate that 32.2% women (the majority was at childbearing age) were overweight and obese [3]. Overweight and obesity during pregnancy is associated with an increased risk of a number of adverse consequences for both mother and baby [4-6]. Prepregnancy body mass index (BMI) and weight gain during gestation are 2 of the most important risk factors for gestational diabetes mellitus (GDM) [7-9]. However, attempts at reducing complications during pregnancy among overweight or obese pregnant women have generally been unsuccessful. An alternative strategy is to take a low glycemic index (LGI) diet. The concept of glycemic index (GI) was developed by Jenkins et al in 1981 as a method of ranking the postprandial glycemic response to equivalent portions of carbohydrate in different foods [10], GI ≤55 was considered as low GI [11]. LGI foods produce lower postprandial increases in blood glucose and reduce diurnal postprandial glucose and insulin responses compared with high GI foods [11,12]. Studies involving women with GDM have shown that an LGI diet reduces postprandial glucose values and the need of insulin [13,14]. However, trials in overweight pregnant women have mostly small sample size and tend to focus on weight change of mothers and babies [15]. Insulin resistance has been accepted as a common underlying basis and indicator of cardiovascular risk. Evidence of effectiveness of the LGI diet during gestation on insulin resistance is needed.

### Objective

The current trial aimed to test the hypothesis that individualized LGI dietary consultation started from the first antenatal visit would reduce the level of insulin resistance on overweight pregnant women and their babies compared with standard diet consultation according to national nutrition recommendations for pregnancy.

## Methods

This study was a randomized, single-blinded controlled intervention trial, with a 1:1 allocation ratio, and approved by the Ethics Committee of Children’s Hospital of Fudan University (approval NO.071-2012). Written informed consent was obtained from every subject before baseline data collection. The trial was registered at the ClinicalTrials.gov registry (NCT01628835).

### Subjects

Overweight or obese pregnant women were assessed for eligibility and recruited from primary antenatal care settings of Kunshan Maternity and Child Care Center (Kunshan city, Jiangsu province, China) and the International Peace Maternity and Child Health Hospital of China Welfare Institute (Shanghai, China) from June 2012 to October 2015. Women were eligible if they met all of the following criteria: first antenatal visit ≤16 weeks of gestation (changed from ≤14 weeks in registration), aged 18 to 45 years, with BMI ≥24 kg/m^2^, and would take routine prenatal examinations. Exclusion criteria were artificial impregnation, a history of hypertension, diabetes, and coronary heart disease, or subjects with mental disorder or special dietary needs (eg, vegetarianism). Subjects were recruited from the early pregnancy clinic in the hospitals where health care records of pregnant women were established. One obstetrician was trained for each center and assigned to judge the eligibility, complete the informed consent process, and implement random allocation according to the randomization plan. Subjects were referred to either one of the 2 dietitians to receive different diet consultations but were blinded to the content of the intervention.

### Randomization and Dietary Intervention

The randomization sequence was generated, and block randomization (block size=4) was performed in the center by a statistician, using Microsoft Office Excel. A 1:1 randomized allocation plan for 200 subjects was provided to each center followed by a training of standard manipulation to the obstetrician who would be in charge of the allocation.

All the participants in the 2 arms received a standard nutrition and physical activity consultation according to the national recommendations of the Chinese Nutrition Society [16], as well as advice to keep gestational weight gain (GWG) according to the 2009 Institute of Medicine guidelines [17]. The dietary consultation consisted of individualized diet assessment, followed by diet planning. Participants were first asked to recall food consumption in 24 hours of the nearest working day for diet assessment, on the basis of which daily intake of conventional nutrition such as total energy, protein, fat, and carbohydrate intake was provided. Then, the dietitian worked with the participant to make an individualized diet plan to the meet the standard goals. The difference of consultation in the experimental group was the additional diet GI and glycemic load (GL) calculations in the diet assessment, and a diet plan was made to achieve an LGI goal with consideration of individual food preference. A mobile phone app DietGI (Children’s Hospital of Fudan University, Multimedia Appendix 1) was equipped with the function of selecting food types and amount of intake for every meal; the GI and GL were output for a single meal or 3 meals a day. In addition, by using the tool, the dietitian demonstrated how to adapt foods for 3 meals to achieve an LGI diet, including replacing high GI foods with LGI foods that they preferred with the preferred portion size. The knowledge of combining foods in meals and cooking techniques (eg, lessen cooking time) to achieve lower GI was also provided. We developed a customized excel worksheet as the tool for quantitative calculation of diet GI and GL, which was designed with algorithms following the international rules and the published GI information of Chinese [8] and international foods [18-20]. The 24-hour diet records and nutrition assessments at 3 visits were saved in the worksheet for every subject. To blind participants to dietary assignment and avoid contamination, dietary consultation was arranged on a different day and by separate dietitians.

In total, 3 diet consultation interviews were incorporated in the routine antenatal care to avoid loss of follow-up at first antenatal visit, middle gestation visit (24th to 28th week) with the routine 75 g oral glucose-tolerance test (OGTT), and late gestation visit (34th to 36th week) with routine liver and kidney function test. Project nurses contacted and made an appointment with participants before the due visit to increase attendance or answer any dietary queries. Between visits participants were followed up by a telephone interview at least once a month to prompt compliance.

### Data Collection and Outcome Measurements

At the first antenatal visit, demographic characteristics and clinical and anthropometric measures were collected after subjects signed the informed consent. BMI was calculated as weight (kg)/(height [m])^2^ and categorized according to Chinese categories (underweight <18.5 kg/m^2^, normal weight 18.5 to 23.9 kg/m^2^, overweight 24 to 27.9 kg/m^2^, and obese ≥28 kg/m^2^) [21]. The gestational age was estimated from self-reported last menstrual period and corrected by the first routine ultrasound examinations around the 14th week. Maternal GWG was defined as the weight gain from the first antenatal visit to delivery. Routine examinations of plasma glucose, serum insulin, glycosylated hemoglobin (HbA_1c_), and blood pressure (BP) were conducted at the prenatal care setting of each center. The homeostasis model assessment of insulin resistance (HOMA-IR) was calculated as follows [22]: (fasting plasma glucose [mmol/L])×(fasting insulin [mIU/L])/22.5. GDM was defined according to international standards [23,24] based on the routine 75 g OGTT screening between 24 and 28 weeks of gestation. The routine lab examination results were extracted from the hospital information system. Cord blood was collected at birth, and serum was separated and stored in the freezer (−20 degree) for no more than 1 night and transferred to a −80-degree freezer according to standard protocols. The stored cord blood serum from 2 centers was transported to the central lab and were examined for C-peptide levels instead of insulin levels as the index of neonatal beta cell function [25,26].

The primary outcomes included incidence of GDM, maternal insulin levels before delivery, and cord blood C-peptide levels as indicators of maternal and neonatal insulin resistance levels. Maternal secondary outcome measures included GWG, incidence of gestational hypertension, and a cesarean. Key secondary outcomes for the fetus or neonate included birth weight, preterm birth before 37 weeks of gestation, and incidence of macrosomia. Gestational hypertension was defined as a systolic BP of 140 mmHg or more or a diastolic BP of 90 mmHg or more on at least 2 occasions at least 4 hours apart in a patient who was normotensive before 20 weeks of gestation. Low birth weight and macrosomia were considered as birth weight <2500 g or birth weight ≥4000 g, respectively.

### Power

The sample size was calculated based on one of the primary aims: GDM. Considering that there was no analogous research at the time, relative risk was assumed as 3.57 in overweight pregnant women with a baseline risk of 3.6% according to the effect of exercises intervention compared with none [27]. In total, 400 overweight pregnant women in a 1:1 ratio were needed to achieve 90% power to detect the effect size at a 5% significance level, allowing for an expected withdrawal of 10%. For continuous outcomes, a mean difference of 0.3 SD will be powered (power ≥0.85) by this sample size at an alpha of .05.

### Statistical Analysis

Continuous data are reported as mean (SD) or median (interquartile range), and categorical data are reported as percentages. Statistical analyses of primary outcomes were in accordance to the intention-to-treat strategy. Comparisons between the 2 arms were carried out by using independent samples of *t* tests for continuous variables and chi-square tests for categorical variables. Stata version 15.0 was used for all statistical analyses. Results were considered significant when the *P* value was <.05. Spearman correlation analyses were performed to test the relationship between change values of selective outcomes.

## Results

### Baseline Characteristics of Study Subjects

**Figure 1:**
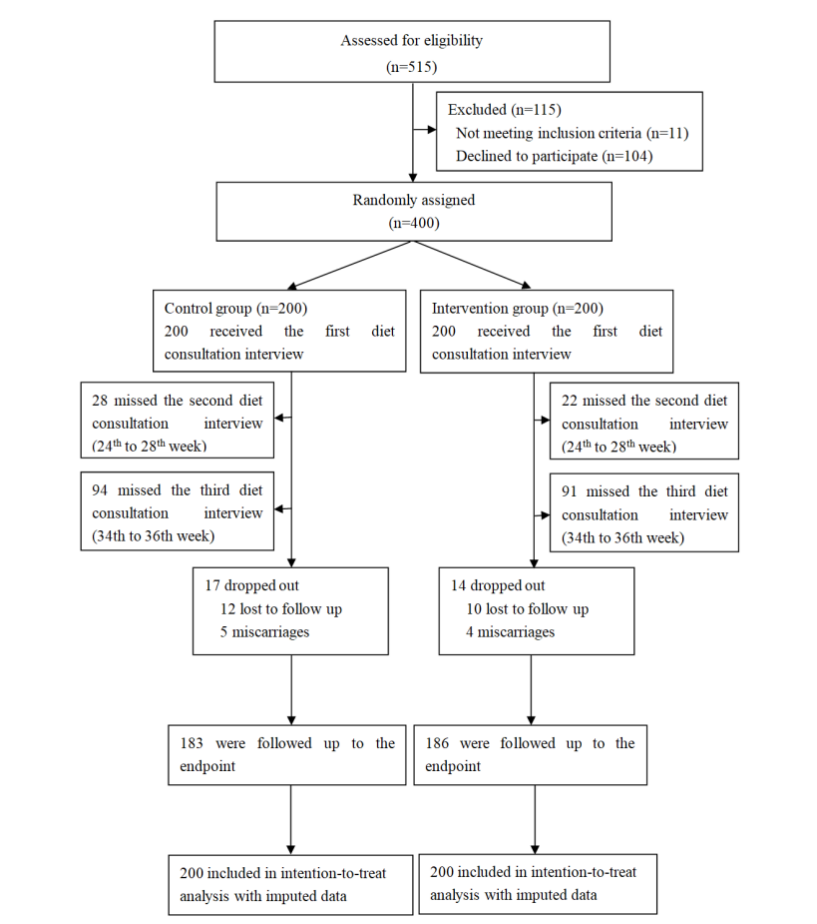
CONSORT flow diagram.

The general characteristics between the intervention group and the control group did not significantly differ (Table 1). The flow of participants is shown in Figure 1. In total, 400 women who signed the informed consent forms were randomly assigned to 2 arms with 200 subjects for each; all received the first diet consultation interview. About 10% of the subjects missed the second diet consultation interview and almost half missed the third visit. Finally, 183 in the control group and 186 subjects in the intervention group remained at the end point with complete birth data and successful biosample collection. The rate of loss to follow-up in the control group was similar to that in the intervention group (8.5% vs 7.0%, respectively; *P*=.58). General characteristics of participants at baseline are presented in Table 1, showing no significant between-group differences.

**Table 1:** Baseline characteristics of the study participants (N=200).

Characteristics	Control group	Intervention group	*P* value
Age (years), mean (SD)	28.0 (3.7)	28.1 (3.6)	.86
Gestational week (week), mean (SD)	12.4 (2.2)	12.2 (2.2)	.49
Height (cm), mean (SD)	162.4 (5.7)	161.7 (6.1)	.26
Weight (kg), mean (SD)	73.9 (9.5)	74.2 (9.0)	.69
Body mass index (kg/m^2^), mean (SD)	28.0 (3.0)	28.4 (3.0)	.19
Systolic blood pressure (mmHg), mean (SD)	120.7 (12.4)	121.1 (11.2)	.70
Diastolic blood pressure (mmHg), mean (SD)	74.6 (9.9)	74.6 (10.1)	.98
Fasting plasma glucose (mmol/L), mean (SD)	4.7 (0.6)	4.7 (0.4)	.56
Insulin (uU/mL), median (IQR^a^)	9.5 (6.8-13.5)	10.0 (6.6-13.9)	.57
HOMA-IR^b^, median (IQR)	2.0 (1.3-2.7)	2.0 (1.4-3.0)	.59
HbA_1c_^c^ (%), mean (SD)	5.3 (0.8)	5.2 (0.4)	.44

^a^IQR: interquartile range.

^b^HOMA-IR: homeostasis model assessment of insulin resistance.

^c^HbA_1c_: glycosylated hemoglobin.

**Table 2:** Maternal and fetal outcomes (N=200).

Outcomes		Control group	Low glycemic index diet group	*P* value
**Maternal outcomes at delivery**				
	Insulin (uU/mL), median (interquartile range)	12.8 (10.5-12.8)	12.8 (9.3-13.2)	.18
	Insulin^a,b^ (uU/mL), mean (SD)	1.5 (7.8)	1.9 (6.6)	.56
	Gestational diabetes mellitus, n (%)	43 (21.5)	45 (22.5)	.33^b^
	Gestational hypertension, n (%)	32 (16.2)	45 (22.8)	.11^b^
	Weight (kg), mean (SD)	85.3 (10.3)	84.0 (10.6)	.23^b^
	Gestational weight gain (kg), mean (SD)	11.2 (6.3)	9.6 (7.4)	.02
	Gestational age (week), mean (SD)	39.8 (1.7)	39.7 (1.2)	.33
	Caesarean, mean (SD)	107 (58.5)	124 (67.0)	.09
	HbA_1c_ (%), mean (SD)	5.4 (0.4)	5.4 (0.3)	.79
	HbA_1c_^a,b,c^ (%), mean (SD)	0.10 (0.75)	0.2 (0.4)	.35
**Neonatal outcomes**				
	Cord blood C-peptide (ng/mL), mean (SD)	0.85 (0.61)	0.86 (0.67)	.84
	Birth weight (g), mean (SD)	3452.5 (527.1)	3513.86 (522.4)	.26
	Macrosomia, n (%)	21 (19.0)	31 (23.0)	.15
	Preterm, n (%)	23 (11.7)	18 (8.7)	.11

^a^Median (interquartile range), *P* values based on log-transformed values.

^b^Levels in third trimester minus levels at baseline.

^c^HbA_1c_: glycosylated hemoglobin.

### Outcomes

As shown in Table 2, participants in the intervention group gained less body weight than those in the control group (9.6 [SD 7.4] vs 11.2 [SD 6.3], respectively; *P*=.03). We did not observe significant differences in maternal insulin levels at late gestation, change from baseline, and cord blood C-peptide, which were examined as markers of insulin resistance.

### Diet Assessments

The dietary intakes of nutrition at 3 visits and changes across 3 visits during gestation are shown in Table 3. At the baseline, the second visit, and the last visit, no significant differences in total intake of energy, protein, fat, carbohydrate, diet GL, or GI were observed, whereas at the third visit, greater fiber intakes were observed in the LGI diet group compared with the control group (11.6 (SD 8.0) g vs 8.9 (SD 5.6) g, respectively; *P*=.006). The overall increment of diet fiber intakes in the LGI diet group significantly differs from the overall decrease in the control group across the intervention period (*P*=.006). Although we did not find significantly different responses of diet GI between the 2 intervention groups, we found that steady reductions of diet GL in both groups (15.2 (SD 76.8); *P*=.004), which includes both GI and amount of foods in calculation, are in line with the observed reduction of diet carbohydrate intake (−25.0 [SD 107.1]; *P*=.007). The posthoc analysis revealed that change of diet GI (levels at late gestation minus levels at baseline) was weakly correlated with changes of maternal insulin levels (r=0.19; *P*=.02), the significance remained after adjustment of maternal age and GWG.

The situation of adherence to interventions and missing data were similar between the 2 groups (Table 4), indicating a minor chance of confounding bias.

**Table 3:** Dietary intake of glycemic load (GL), glycemic index (GI), and nutrients (per day) at 3 visits and changes.

Diet assessments		Control group^a^, mean (SD)	Low glycemic index (LGI) diet group^b^, mean (SD)	*P* value
**GL**				
	Baseline	130.6 (59.6)	132.4 (53.5)	.75
	Second trimester	124.1 (55.4)	125.1 (49.4)	.85
	Third trimester	111.6 (59.4)	112.4 (56.0)	.91
	Change^c^	−14.4 (79.1)	−16.0 (74.8)	.88
**GI**				
	Baseline	64.1 (9.7)	64.5 (8.7)	.69
	Second trimester	62.8 (9.8)	64.8 (9.4)	.05
	Third trimester	64.3 (10.4)	63.2 (10.4)	.42
	Change	0.25 (13.7)	−1.1 (12.6)	.46
**Energy (kcal)**				
	Baseline	1488.8 (522.3)	1522.8 (472.3)	.49
	Second trimester	1539.2 (561.9)	1515.2 (543.9)	.69
	Third trimester	1337.0 (495.0)	1373.4 (508.6)	.60
	Change	−125.9 (660.0)	−120.8 (682.2)	.96
**Carbohydrate (g)**				
	Baseline	203.1 (87.2)	203.6 (72.0)	.95
	Second trimester	195.8 (79.7)	191.9 (70.6)	.63
	Third trimester	171.4 (83.5)	173.3 (75.0)	.86
	Change	−25.0 (107.0)	−24.7 (112.6)	.97
**Protein (g)**				
	Baseline	60.93 (25.33)	60.27 (24.45)	.79
	Second trimester	66.88 (28.71)	63.61 (25.81)	.26
	Third trimester	63.00 (26.41)	61.89 (26.44)	.76
	Change	−0.41 (34.4)	−1.0 (32.2)	.88
**Fat (g)**				
	Baseline	48.1 (27.8)	52.1 (26.0)	.14
	Second trimester	53.8 (33.1)	52.70 (30.5)	.75
	Third trimester	44.8 (24.4)	47.7 (33.3)	.47
	Change^c^	−2.5 (30.9)	−2.3 (42.4)	.97
**Fiber (g)**				
	Baseline	11.1 (8.0)	10.2 (6.7)	.19
	Second trimester	11.8 (8.1)	11.1 (6.8)	.41
	Third trimester	8.9 (5.6)	11.6 (8.0)	.006
	Change^c^	−2.4 (10.6)	1.5 (9.8)	.005

^a^Control group: n=200, 172, and 106 at baseline, second trimester, and third trimester, respectively.

^b^LGI diet group: n=200, 178, and 109 at baseline, second trimester, and third trimester, respectively.

^c^Change: levels in third trimester to levels at baseline.

**Table 4:** Comparisons of adherence to dietary interventions and missing data in the low glycemic index (LGI) diet group and the control group (N=200).

Descriptions			Control group, n (%)	Low glycemic index diet group, n (%)	*P* value
**Adherence to dietary interventions**					
	At least 2 times		181 (90.5)	186 (93.0)	.36
	All 3 times		106 (53.0)	109 (54.5)	.76
**Missing data at end point**					
	**Maternal outcomes**				
		Weight	17 (8.5)	14 (7.0)	.58
		Insulin	85 (42.5)	77 (38.5)	.42
		HbA_1c_^a^	93 (46.5)	84 (42.0)	.37
	**Neonatal outcomes**				
		Birth weight	17 (8.5)	15 (7.5)	.71
		Cord blood C-peptide	107 (53.5)	100 (50.0)	.48

^a^HbA_1c_: glycosylated hemoglobin.

## Discussion

### Principal Findings

This trial was conducted in real-world clinical practice. Starting from early gestation, 3 individualized LGI diet consultations provided by a clinical dietician by using a diet GI and GL calculator failed to make a significant difference in maternal or neonatal insulin levels in overweigh and obese pregnant women compared with standard diet counseling. However, some changes in diet habits in the intervention group were observed that were different from those in the control group, including more fiber intake and less carbohydrate intake but comparable total energy intake, which are favorable to achieving lower diet GI.

### Limitations

Our study has certain limitations. First, compliance to intervention in the 2 groups is lower than expected. The attendance to diet intervention interview remained over 90% at the second visit but dropped to 53.8% at the third visit. About 5% of the missed subjects were because of miscarriage and 5% because of moving to a different place for delivery; the overall rate is under assumption in sample size planning. The proportions of dropout are similar in the 2 arms; under the assumption of missing at random, missing values of primary outcomes are not likely to bias the comparisons, whereas this may lower the statistical power. A second explanation to poor compliance may be the fact that changing the diet habit is even harder for pregnant Chinese women, whose diet and nutrition status receive more attention from families. More intensive interactions with dietitians or convenient diet management tools, for example, a diet GI calculator, may help improve the overall compliance. In this trial, the diet GI calculated is performed by a researcher. If the tool was available to each subject and could be used every day, the compliance to a healthy LGI diet would be greatly improved. Second, nutrition intakes may be underestimated in this trial. Total energy intake was assessed by a 24-hour food record, which is lower than the recommendations for pregnant women by the Chinese Nutrition Society (1500 vs 2300 kcal/day) [16]. This may be due to systematic underestimation of the amounts of food intake by the 24-hour diet recall; however, this situation is equally distributed in the 2 arms and will not likely bias the comparisons. Moreover, to make recruitment easier, the gestational week for enrollment was extended to 16 weeks instead of ≤14 weeks in the trial registry. We think this change will not bias the main findings of the study. Finally, physical activity of subjects is not recorded.

### Comparison With Previous Work

This study is one of a few trials to examine the effect of LGI diet intervention on insulin resistance of overweight pregnant women and their newborns. The intervention for the active intervention group is 3 individualized diet GL assessments and consultations using a mobile phone app starting at early gestation without providing any foods. The primary aim is to assess additional effectiveness of LGI diet intervention on maternal and neonatal insulin resistance levels compared with the conventional standard diet consultation according to the national nutrition recommendations for pregnant women.

We did not observe significant differences in maternal insulin levels at late gestation, change from baseline, or cord blood C-peptide, which is examined as markers of neonatal insulin resistance. These findings are contrary to our hypothesis, indicating that the standard diet consultation plus LGI diet intervention does not make significant additional improvement in maternal and neonatal insulin resistance. Insulin resistance level changes during gestation are not commonly measured as primary outcomes in previous LGI intervention trials. One study by Walsh et al conducted in a subgroup of low glycemic index diet in pregnancy to prevent macrosomia The Randomised cOntrol trial of Low (ROLO) study [28] reported some benefits of LGI diet consultations initiated from early gestation to maternal insulin resistance [29]. Participants in the LGI intervention group also gained less weight by 1.3 kg than those in the control group [28]; the effect size is similar to what we observed in our study (1.6 kg). However, controls of the ROLO study received routine antenatal care, involving no formal dietary advice about GWG. In our study, the standard diet consultation received in the active comparative control arm may be too effective and make it hard to make a significant difference from the LGI group. This can be an important explanation to the negative findings in most of the outcomes. This is supported by the finding that the maternal insulin-level changes of the control group of our study are very close to the changes in the intervention group of the previous study (1.5 (SD 7.8) vs 1.79 (SD −2.6 to −4. 6), respectively) [28]. A number of studies have investigated the effect of LGI diet in different subjects, such as in pregnant women with gestational hyperglycemia [30], with GDM [13,31], women at high risk of GDM [32], or in healthy women [33]; clinical outcomes such as birth weight, incidence of GDM, insulin medication use, and GWG are examined. Evidence on the effects of LGI diet in overweight and obese pregnancies is limited. In total, 1 relevant trial is conducted in 46 overweight or obese pregnant women to compare the effect of an LGI diet with a low-fat diet [15]. Except for diet counseling, the interventions also provided LGI foods, such as carbohydrate-rich foods, fats, and snacks. However, they did not find any significant differences in insulin, HbA_1c_, or HOMA-IR in the 2 groups. The results are compatible with our findings.

One strength of our study is the quantitative diet GI and GL estimation and records along with each visit of diet intervention, which allows us to observe the diet behavior changes. Effectiveness of the LGI intervention is expected to be achieved through change of diet habits, which is measured by food intake of different contents of nutrients. Our findings in diet fiber, carbohydrate, and GL support that these overweight and obese pregnant women have clear but very variable favorable responses to the diet intervention.

Although previous randomized trials show effectiveness and feasibility of using email, internet, or mobile phones in changing prediabetic individuals in changing their lifestyles and biological index [34], changing the lifestyle of overweight subjects by consultation is never an easy goal to achieve; it is even harder with pregnant women. Pregnant Chinese women traditionally are encouraged to eat more food than they need to ensure sufficient nutrition intake to the fetus. A study shows that gaining too much weight is a more critical issue in the Chinese population than other races [35]. We also find that a change of diet GI was weakly correlated with changes of maternal insulin levels, even after adjustment of maternal age and GWG. This result enhances our belief of the long-term healthy effectiveness of adopting the LGI diet for reducing metabolic risk in a high-risk population. The findings and manipulation experiences of the intervention (the longitudinal quantitative assessment of diet GI and low glycemic diet consultations with pregnant women) from this otherwise negative trial may be of interest to patients and clinicians in this field.

### Conclusions

Compared with standard nutrition consultations with overweight or obese pregnant women, 3 individualized LGI diet consultations starting from early gestation do not make a significant difference in maternal or neonatal insulin levels. However, the LGI intervention, which is equipped with accurate diet GI and GL calculation, makes some differences in diet habits among overweight pregnant women, including more fiber intake and less carbohydrate intake, which are known to be favorable to achieve a lower diet GI. The pregnancy period is limited; we encourage to provide LGI diet education and an electronic diet GI calculation tool to acquire more significant effectiveness in reducing insulin resistance among a high-risk population.

## Appendix

Multimedia Appendix 1 Introduction to Diet GI app (in Chinese).

## Abbreviations

BMI: body mass index
BP: blood pressure
GDM: gestational diabetes mellitus
GI: glycemic index
GL: glycemic load
GWG: gestational weight gain
HbA _1c_: glycosylated hemoglobin
HOMA-IR: homeostasis model assessment of insulin resistance
LGI: low glycemic index
OGTT: oral glucose-tolerance test
ROLO: Randomised cOntrol trial of LOw
