# Supplementary material for: Effectiveness of Low Glycemic Index Diet Consultations Through a Diet Glycemic Assessment App Tool on Maternal and Neonatal Insulin Resistance: A Randomized Controlled Trial
Source: JMIR Mhealth Uhealth. 2019 Apr 18;7(4):e12081. doi: 10.2196/12081 (PMC6503641; doi:10.2196/12081)
Supplement: Multimedia Appendix 1 [file mhealth_v7i4e12081_app1.pdf]

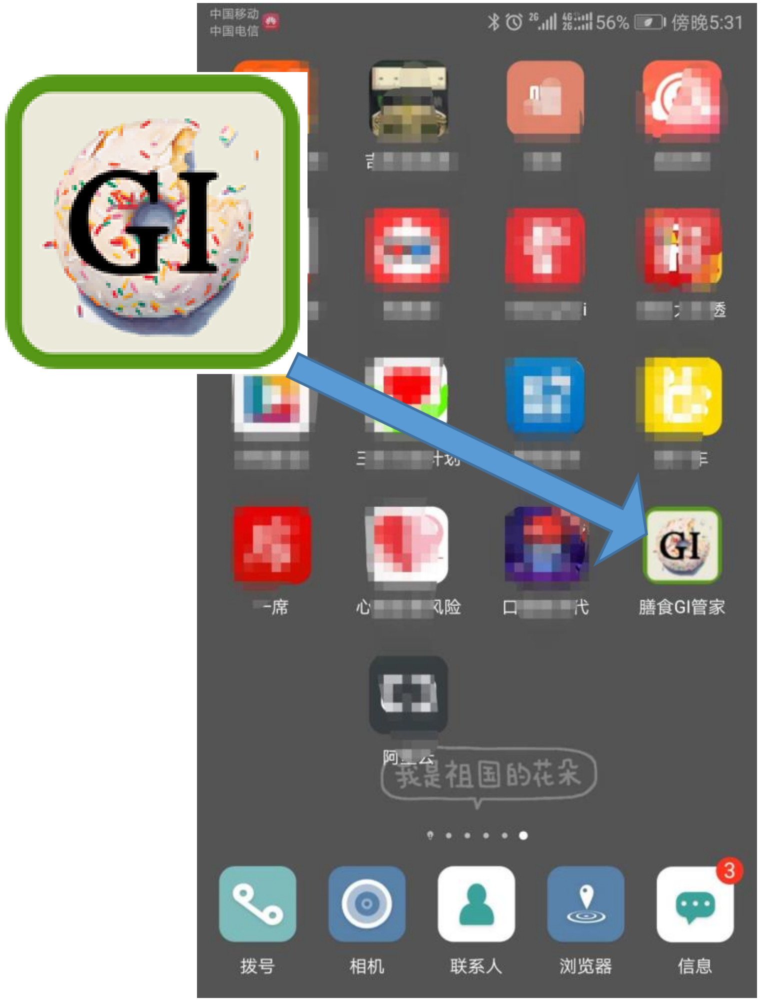

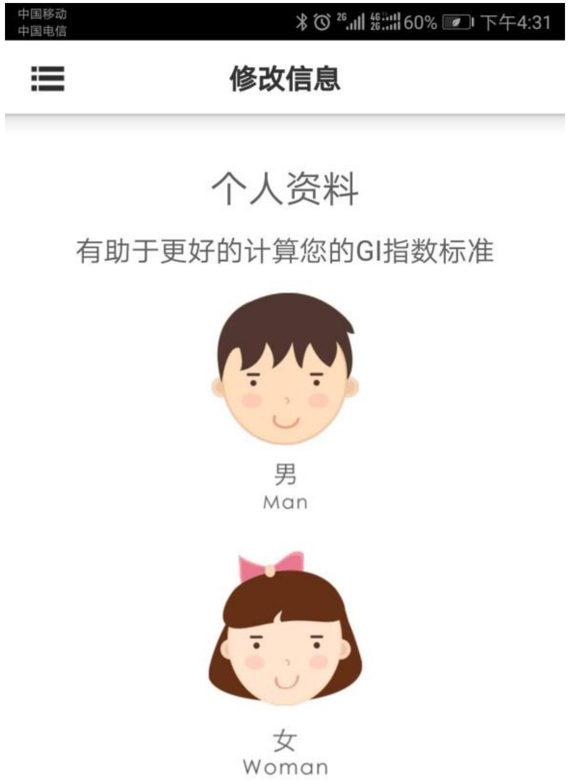

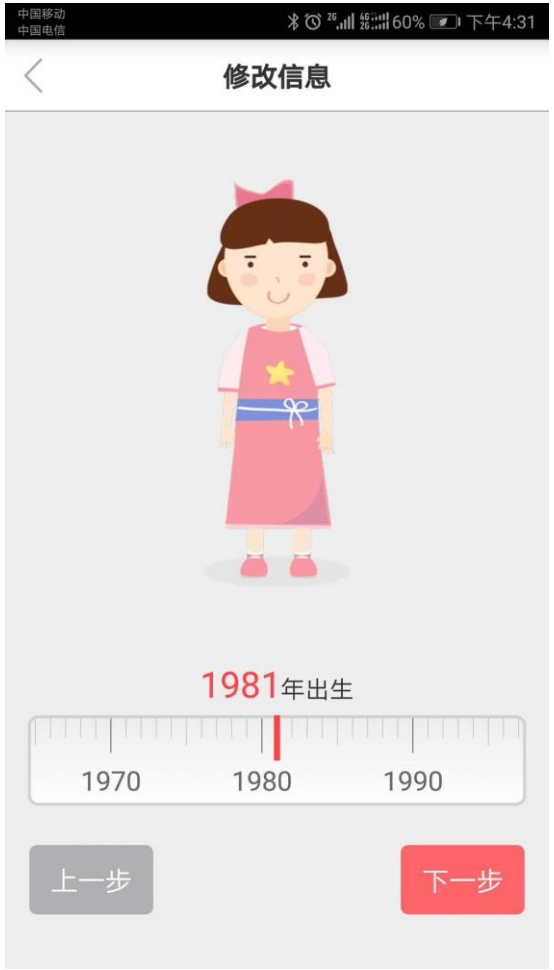

Enter personal information

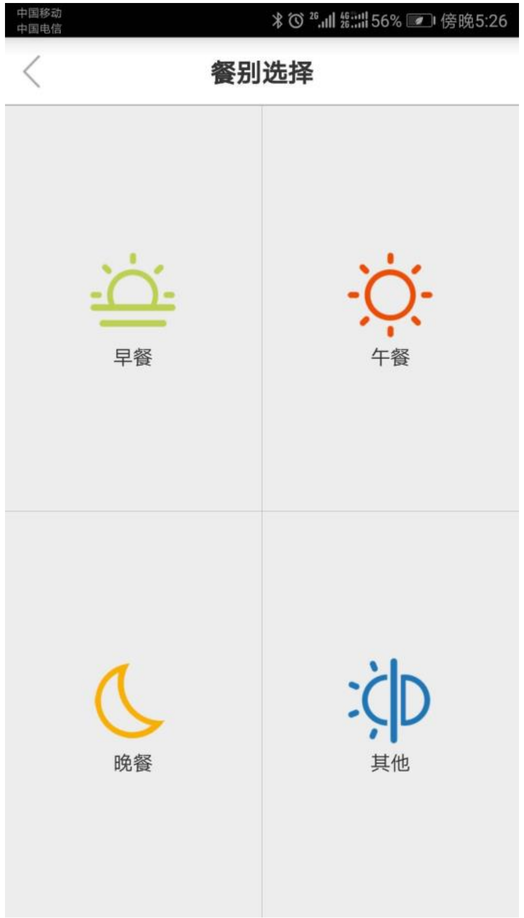

Select your breakfast/lunch/supper/snacks

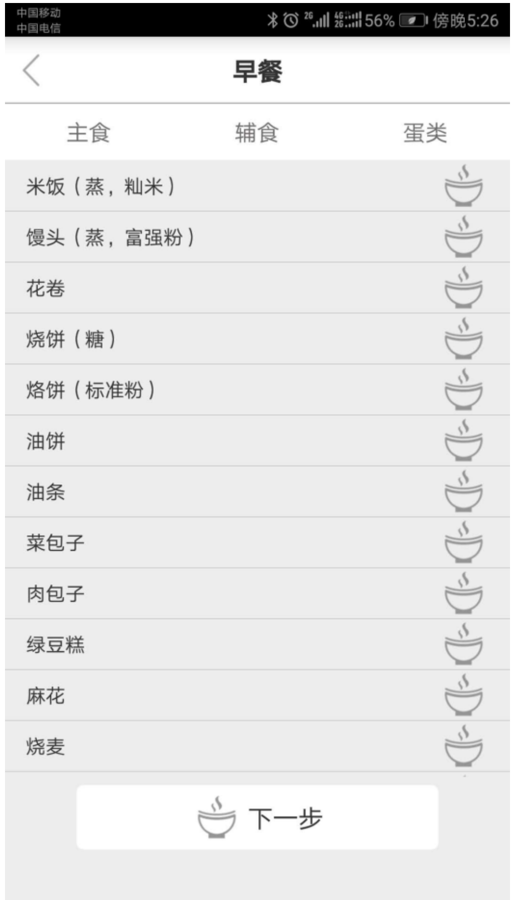

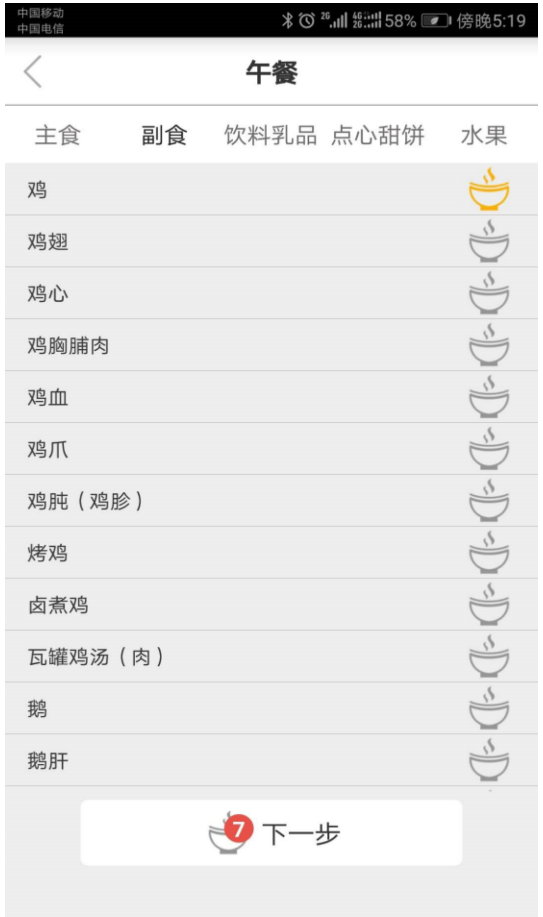

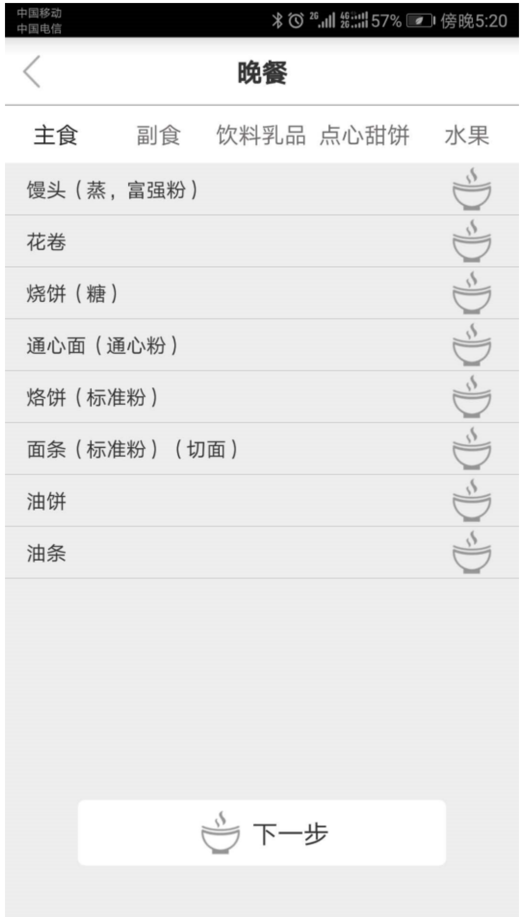

Input the type and amount of food intake

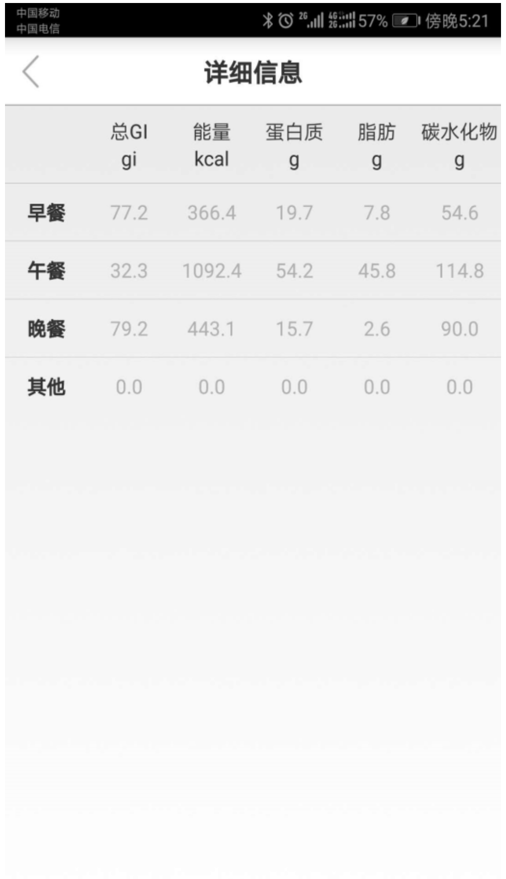

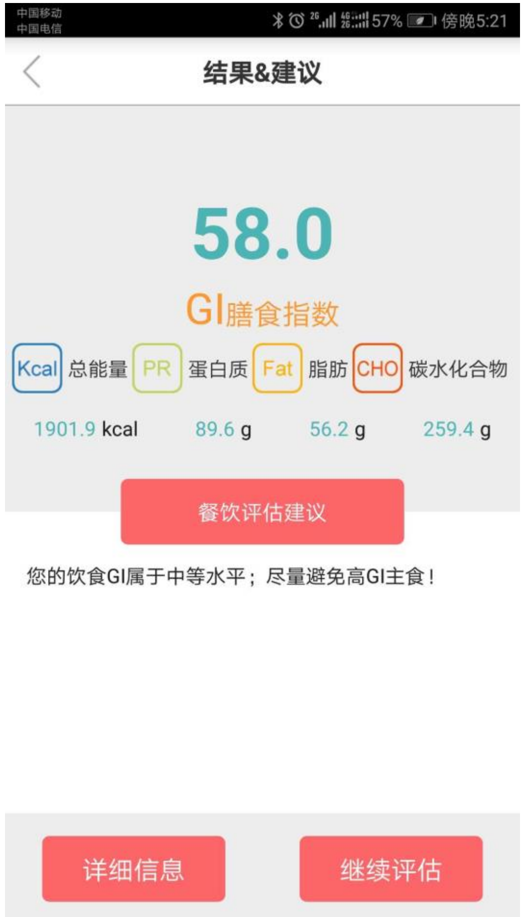

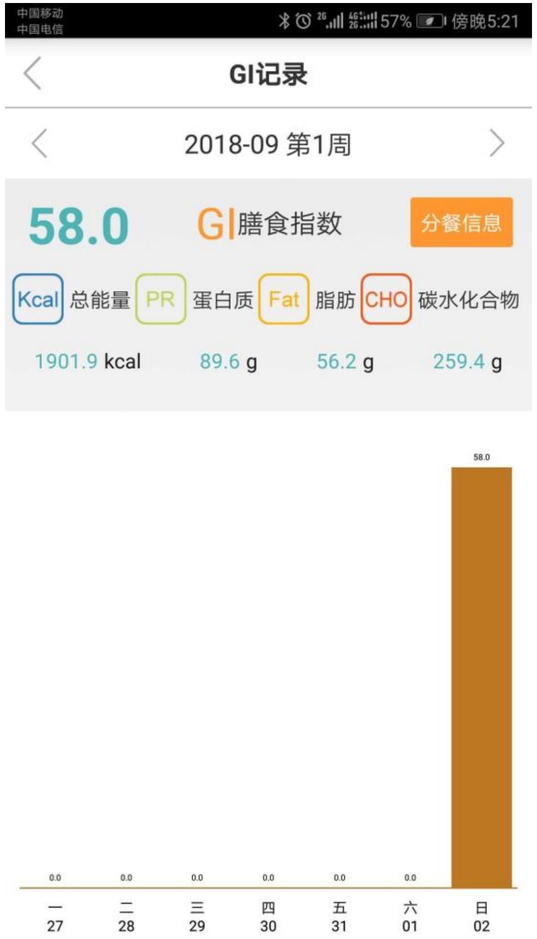

Calculate and output
